# A Novel Complex-Valued Gaussian Measurement Matrix for Image Compressed Sensing

**DOI:** 10.3390/e25091248

**Published:** 2023-08-22

**Authors:** Yue Wang, Linlin Xue, Yuqian Yan, Zhongpeng Wang

**Affiliations:** School of Information and Electronic Engineering, Zhejiang University of Science and Technology, Hangzhou 310023, China; 222108855022@zust.edu.cn (Y.W.); 119029@zust.edu.cn (L.X.); 222108855053@zust.edu.cn (Y.Y.)

**Keywords:** compressed sensing, measurement matrix, Gaussian matrix, Gram–Schmidt orthogonalization, sparse matrix

## Abstract

The measurement matrix used influences the performance of image reconstruction in compressed sensing. To enhance the performance of image reconstruction in compressed sensing, two different Gaussian random matrices were orthogonalized via Gram–Schmidt orthogonalization, respectively. Then, one was used as the real part and the other as the imaginary part to construct a complex-valued Gaussian matrix. Furthermore, we sparsified the proposed measurement matrix to reduce the storage space and computation. The experimental results show that the complex-valued Gaussian matrix after orthogonalization has better image reconstruction performance, and the peak signal-to-noise ratio and structural similarity under different compression ratios are better than the real-valued measurement matrix. Moreover, the sparse measurement matrix can effectively reduce the amount of calculation.

## 1. Introduction

Due to the continuous development of the digital age, massive images have become increasingly challenging to store and transmit. To effectively address the transmission of such image data, compressed sensing is widely employed in image transmission to reduce the bandwidth required for transmission [1,2,3]. By combining the process of sampling and compression, compressed sensing can acquire a signal at a lower sampling rate and accurately recover the original signal, thereby substantially reducing the overhead of data acquisition and storage. After long-term development, compressed sensing has found widespread application in numerous domains, including wireless communication [4], medical imaging [5], smart city construction [6], and video codec [7,8]. Compressed sensing theory primarily consists of three components: the sparse representation of the signal, the measurement matrix’s design, and the reconstruction algorithm’s design. How to choose the measurement matrix will directly affect the quality of image reconstruction. At present, the majority of research on measurement matrix design focuses on real-valued measurement matrices, such as Gaussian random matrix [9], Bernoulli matrix [10], and partial Hadamard matrix [11], while research using a complex-valued matrix as a measurement matrix is relatively few. To further enhance image reconstruction quality in compressed sensing, we used two different Gaussian random matrices to construct a complex-valued measurement matrix.

### 1.1. Related Work

In reference [12], the authors first generated a logistic chaotic sequence and then constructed a measurement matrix through the chaotic sequence. The feasibility of the measurement matrix was verified via theory and experimental analysis. In reference [13], the authors proposed a measurement matrix based on a Chebyshev chaotic sequence and proved the feasibility of the proposed measurement matrix. In reference [14], the author proposed using a Chebyshev–Vandermonde-like matrix as the measurement matrix, which also achieved good results. In reference [15], the authors used a Toeplitz matrix and a circulant matrix to construct the measurement matrix. This construction method reduced the difficulty of hardware implementation to a certain extent, but the reconstruction accuracy was generally not high. In reference [16], the authors used the semi-tensor product to construct a measurement matrix, which reduced the storage space required for the measurement matrix and reduced the reconstruction time via parallel reconstruction. In reference [17], a binarized Chebyshev chaotic map and an improved logistic chaotic map were used to construct a mixed chaotic sequence through the XOR operation, thereby improving the reconstructed image’s performance.

Most compressed sensing schemes usually use a real-valued matrix as the measurement matrix. However, in reference [18], the authors used a complex-valued Hadamard matrix instead of a real-valued measurement matrix and implemented the block method to reduce the complexity. The authors found that using the complex-valued measurement matrix resulted in better image reconstruction than the real-valued matrix. In reference [19], the authors proposed using a complex-valued Zadoff–Chu (ZC) matrix as the measurement matrix and employed a block measurement scheme to reduce complexity. The simulation results indicated that the Zadoff–Chu matrix yielded better reconstruction than the real-valued measurement matrix.

### 1.2. Contributions

To enhance the image reconstruction performance in compressed sensing, as inspired by references [18,19], a novel complex-valued measurement matrix was proposed in this work. This work used two Gaussian matrices, which were Schmidt orthogonalized respectively, to form a complex-valued measurement matrix and compared its reconstruction performance with that of the real-valued measurement matrix. It is found that the orthogonal complex-valued Gaussian matrix has better image reconstruction performance.

The main contributions of this work are as follows:

1. We constructed a complex-valued Gaussian matrix as the measurement matrix using two real-valued Gaussian matrices. The results illustrate that the reconstructed image quality is superior when using the complex-valued matrix compared to the real-valued measurement matrix.
2. To enhance the performance of compressed sensing reconstruction, we performed Gram–Schmidt orthogonalization on the two real-valued matrices that make up the complex-valued Gaussian matrix. Based on our experiments, this orthogonalized measurement matrix can significantly enhance the reconstructed image quality.
3. We applied a sparsification operation to the proposed complex-valued measurement matrix to save storage space and reduce the calculation amount during image compression. Our analysis indicates that this sparsification operation effectively reduces the calculation amount required and computational complexity.

## 2. Concepts and Theoretical Basis

### 2.1. Compressed Sensing

In the theoretical model of compressed sensing, if x is a non-sparse signal, the signal x must first be sparsely represented to obtain sparse coefficients. Then, the sparse coefficients are compressed and reconstructed. The process of compression is to project the signal x into a low-dimensional space containing the measurement matrix ϕ of size M×N(M<N) and obtain the measurement y of length M. This process can be expressed using Equation (1):

(1) y=ϕψx=ϕs

In the above equation, s is a sparse vector that is K-sparse on the transform domain ψ. There are only K(K≪N) non-zero numbers, and ${\left\|s\right\|}_{0}=K$. ψ is also called a sparse basis of size N×N.

When the compressed signal is reconstructed, Equation (1) is an underdetermined system with infinite solutions since the length of the measured value y is smaller than the length of the original signal. If one wants to reconstruct the sparse coefficients s from the measurements y, then the sensing matrix θ=ϕψ needs to satisfy the RIP property, which is expressed as follows:

(2) $$(1-\delta_{k}){\left\|s\right\|}_{2}^{2}\leq {\left\|\theta s\right\|}_{2}^{2}\leq (1+\delta_{k}){\left\|s\right\|}_{2}^{2}$$

where $0<\delta_{k}<1$, k represents the number of non-zero elements of the sparse coefficient s. Only when the above properties are satisfied that the reconstruction algorithm can reconstruct the original signal with high probability. The mathematical model of the reconstructed signal is as follows:

(3) $$\hat{s}\;=\arg\min{\left\|s\right\|}_{0}s.t.\;y=\theta s$$

Equation (3) is an $l_{0}$ optimization problem. However, the $l_{0}$ optimization problem is an NP-complete problem. To solve it, it can be transformed into an easy-to-solve $l_{1}$ optimization problem, or to solve the problem by approaching $l_{0}$. After calculating $\hat{s}$ according to the above equation, we can obtain the reconstructed image signal $\hat{x}$ by computing $\hat{x}=\psi^{-1}\hat{s}$. At present, some commonly used reconstruction algorithms include the OMP algorithm [20], SAMP algorithm [21], SL0 algorithm [22], and ISTA algorithm [23]. For reconstruction, we utilized the SL0 algorithm in this work, which reconstructs the image by approximating the $l_{0}$ norm.

### 2.2. Gaussian Random Matrix

Gaussian random matrix is a widely employed measurement matrix in compressed sensing. The design method of this matrix is to construct a matrix of size M×N so that each element of this matrix independently follows a Gaussian distribution with a mean of zero and variance 1/M as follows:

(4) $$\phi ∼N(0,\frac{1}{M})$$

Gaussian random measurement matrix is highly random. It can satisfy the RIP condition with a high probability when the number of measurements M satisfies the inequality M≥cKlog(N/K), where c is a small positive constant and K is the signal sparsity [24]. Therefore, it is highly probable that the compressed image can be reconstructed using a Gaussian random measurement matrix.

### 2.3. Gram–Schmidt Orthogonalization

Gram–Schmidt orthogonalization is a classical basis orthogonalization method [25]. Its basic idea is to use the projection principle to construct a new orthogonal basis based on the existing orthogonal basis. Let $V^{n}$ be an n-dimensional Euclidean space, α and β be the vectors in $V^{n}$, while (α,β) denotes as the inner product of α and β, the projection of α onto β is $proj_{\beta }\alpha =\frac{\left(\alpha ,\beta \right)}{\beta ,\beta }\beta$, and the subspace spanned by the vector group $\alpha_{1},\alpha_{2},\cdots ,\alpha_{m}$ is $\mathrm{span}\left\{\alpha_{1},\alpha_{2},\cdots ,\alpha_{m}\right\}$. Let $\alpha \in V^{n}$, $V^{m}$ be an m-dimensional subspace of $V^{n}$ with orthonormal bases $\alpha_{1},\alpha_{2},\cdots ,\alpha_{m}$, and α not in $V^{m}$. Based on the projection principle, the difference between α and its projection onto the $V^{m}$ subspace is as follows:

(5) $$\beta =\alpha -\sum_{i=1}^{m}{\mathrm{proj}}_{\alpha }\alpha_{i}=\alpha -\sum_{i=1}^{m}(\alpha ,\alpha_{i})\alpha_{i}$$

Moreover, β is orthogonal to the subspace $V^{m}$, that is, orthogonal to the orthogonal basis $\alpha_{1},\alpha_{2},\cdots ,\alpha_{m}$. In this case, β can be obtained via unitization as follows:

(6) $$\alpha_{m+1}=\frac{\beta }{\left|\beta \right|}=\frac{\beta }{\sqrt{\left(\beta ,\beta \right)}}$$

Then, $\alpha_{1},\alpha_{2},\cdots ,\alpha_{m},\alpha_{m+1}$ is the orthonormal basis of the subspace $\mathrm{span}\left\{\alpha_{1},\alpha_{2},\cdots ,\alpha_{m}\right\}$ that is extended by $V^{m}$ on α.

According to the above analysis, for $\mathrm{span}\left\{\alpha_{1},\alpha_{2},\cdots ,\alpha_{m}\right\}$, by starting from the one-dimensional subspace spanned by one of the vectors and repeating the above process of expanding the construction of an orthogonal basis, a set of orthogonal bases for $V^{m}$ can be obtained, which is the idea of Schmidt’s orthogonalization. Schmidt orthogonalization proceeds as follows for m linearly dependent vectors $\alpha_{1},\alpha_{2},\cdots ,\alpha_{m}$ of n-dimensional Euclidean space:

(1) Orthogonalization
  (7) $$\beta_{1}=\alpha_{1}$$
  (8) $$\beta_{2}=\alpha_{2}-\frac{\left(\alpha_{2},\beta_{1}\right)}{\beta_{1},\beta_{1}}\beta_{1}$$
  (9) $$\beta_{3}=\alpha_{3}-\frac{\left(\alpha_{3},\beta_{1}\right)}{\beta_{1},\beta_{1}}\beta_{1}-\frac{\left(\alpha_{3},\beta_{2}\right)}{\beta_{2},\beta_{2}}\beta_{2}$$
  (10) $$\beta_{m}=\alpha_{m}-\frac{\left(\alpha_{m},\beta_{1}\right)}{\beta_{1},\beta_{1}}\beta_{1}-\frac{\left(\alpha_{m},\beta_{2}\right)}{\beta_{2},\beta_{2}}\beta_{2}-\cdots -\frac{\left(\alpha_{m},\beta_{m-1}\right)}{\beta_{m-1},\beta_{m-1}}\beta_{m-1}$$
(2) Unitization

(11) $$\eta_{1}=\frac{\beta_{1}}{\left|\beta_{1}\right|},\eta_{2}=\frac{\beta_{2}}{\left|\beta_{2}\right|},\cdots ,\eta_{m}=\frac{\beta_{m}}{\left|\beta_{m}\right|}$$

The above steps can provide a set of orthogonal bases $\beta_{1},\beta_{2},\cdots ,\beta_{m}$ and orthonormal bases $\eta_{1},\eta_{2},\cdots ,\eta_{m}$ on $\mathrm{span}\left\{\alpha_{1},\alpha_{2},\cdots ,\alpha_{m}\right\}$.

## 3. Compressed Sensing Scheme

### 3.1. Design of Measurement Matrix

To construct an orthogonal complex-valued Gaussian measurement matrix, we started by generating two Gaussian matrices with a mean of zero and a variance of one. We then applied the Gram–Schmidt orthogonalization to each matrix to orthogonalize them. One matrix was used as the real part, and the other was used as the imaginary part. Here, the real part of the measurement matrix was generated as an example, and the imaginary part was generated in the same way. The pseudocode is described below, where Mtx represents the Gaussian matrix, Mtx_orth represents the matrix after Gram–Schmidt orthogonalization, n represents the number of columns of Mtx, and (:, i) represents the ith column of the matrix. The primary step is to iterate over the first i−1 columns, compute the projection of the current column with each of the previous columns, and subtract the projection from the current column to eliminate the correlation and make them orthogonal (Algorithm 1).

**Algorithm 1**: Generate the real part of complex-valued measurement matrixInput: The size of measurement matrixOutput: The real part of the complex-valued measurement matrix  1  Begin  2    Initialize a Gaussian random matrix Mtx  3    Initialize an all-zero matrix Mtx_orth  4    Mtx_orth(:, 1) = Mtx(:, 1)  5    for i = 2 to n do  6      for j = 1 to i − 1 do  7        Mtx_orth(:, i) = Mtx_orth(:, i) − dot(Mtx(:, i), Mtx_orth(:, j))/           dot(Mtx_orth(:, j), Mtx_orth(:, j)) * Mtx_orth(:, j)  8      end  9      Mtx_orth(:, i) = Mtx_orth(:, i) + Mtx(:, i);  10     end  11  end

The above method can construct the complex-valued measurement matrix by adding two orthogonal Gaussian matrices. In reference [26], the authors used Gram–Schmidt orthogonalization to optimize real-valued Gaussian random matrices. In the process of Gram–Schmidt orthogonalization, each time the $\beta_{i}$ vector is calculated, the projection of the first i−1 vectors on $\beta_{i}$ is subtracted from the current vector $\alpha_{j}$. That is, the information of other column vectors is subtracted. After this operation, the independence of the column vector $\beta_{i}$ is gradually **enhanced**. For the measurement matrix, this method can maximize the incoherence of the column vectors to improve the performance of compressed sensing reconstruction.

Compressed sensing can be regarded as extracting features from images for compression purposes. This work constructed a complex-valued measurement matrix using two orthogonal Gaussian matrices to achieve this. Using this measurement matrix for image compression is equivalent to extracting double the number of image features of a real-valued measurement matrix. This approach provides more comprehensive image information, resulting in better reconstruction performance.

### 3.2. Compressed Sensing with Complex-Valued Measurement Matrix

**Figure 1:**
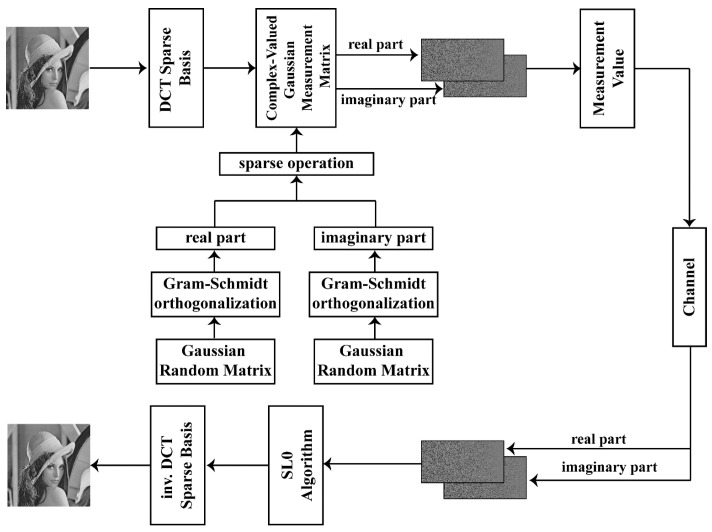
Framework of the compressed sensing scheme.

Below is an overview of our proposed image-compressed-sensing scheme, which uses a complex-valued measurement matrix. The diagram below shows the framework of the scheme (Figure 1):

Step 1: Image sparsification. Use Discrete Cosine Transform (DCT) to make the image sparse.
Step 2: Construct the measurement matrix. Two Gaussian matrices are orthogonalized via Gram–Schmidt orthogonalization, respectively. Then, one is used as the real part of the measurement matrix, and the other is used as the imaginary part.
Step 3: Image compression. Apply the measurement matrix to compress the image.
Step 4: Image reconstruction. Use the SL0 algorithm to reconstruct the sparse signal of the original image in the DCT domain.
Step 5: Inverse sparsification. Use inverse sparsification operation to obtain the original image from the sparse signal in the DCT domain.

## 4. Experimental Analysis

**Figure 2:**
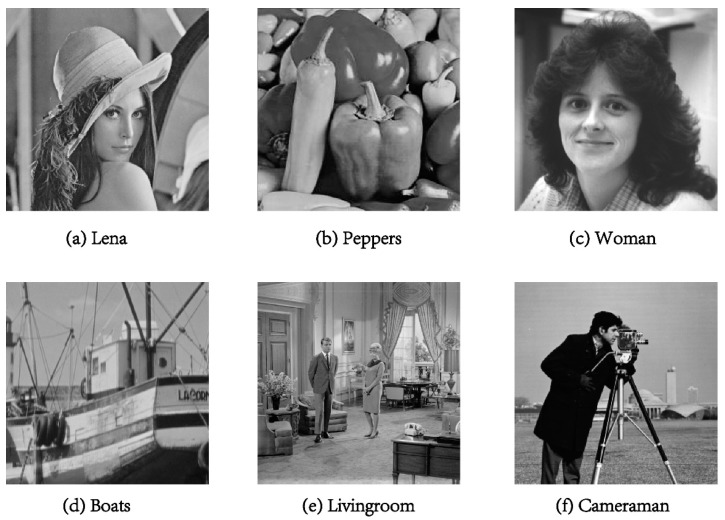
Experimental images.

This work implemented all simulation experiments via Matlab2021a, using a computer with Intel Core i5-6300HQ CPU at 2.30 GHz, 12 GB memory, and Windows 10 operating system. We chose images of various sizes for the experiment and used DCT as the sparse basis. The reconstruction algorithm we utilized was the SL0 [22] algorithm; the source code can be obtained from the author’s home page (http://ee.sharif.ir/~SLzero/ (accessed on 10 July 2023), and the sigma_min was set to 0.01. In the following section, M and N denote the original image’s size, and if the compression ratio (CR) is mentioned in the description, M is the size of the compressed image, that is, M=CR×M. To compare the performance of the reconstructed images, in this work, the Gaussian matrix [9], Bernoulli matrix [10], partial real-valued Hadamard matrix [11], mixed chaotic–Bernoulli (MB) matrix [17], sequency-ordered complex Hadamard transform (SCHT) matrix [27], Zadoff–Chu matrix [19], and orthogonal complex-valued Gaussian matrix were used as measurement matrices for comparison. The images used in the experiments are shown in Figure 2.

### 4.1. Peak Signal-to-Noise Ratio Analysis

The peak signal-to-noise ratio [28] (PSNR) is a commonly utilized standard for evaluating the quality of image reconstruction. Essentially, a higher PSNR value means that the difference between the original and reconstructed images is smaller, which translates to greater accuracy in reconstruction. The formula for calculating the PSNR for image reconstruction evaluation is as follows:

(12) $$PSNR=10\mathrm{lg}\frac{255\times 255}{(\frac{1}{M\times N})\sum_{i=1}^{M}\sum_{j=1}^{N}{\left(X(i,j)-Y(i,j)\right)}^{2}}$$

where X(i,j) is the value of the pixel at the corresponding position of the original image, and Y(i,j) is the value of the pixel at the corresponding position of the reconstructed image.

We compared our proposed measurement matrix to other commonly used ones to analyze its performance. Specifically, we evaluated the effectiveness of our complex-valued measurement matrix by comparing it to several other matrices, including the Gaussian random measurement matrix, Bernoulli matrix, partial real-valued Hadamard matrix, MB matrix, SCHT matrix, and ZC matrix when the compression ratio was 0.2 and 0.5. To ensure accuracy, we conducted ten experiments and averaged the results, which are presented in Table 1 and Table 2. The tables show the reconstruction performance of the “Lena”, “Peppers”, “Woman”, and “Boats” images. From the results in Table 1, we observed that the complex-valued Gaussian matrix performed similarly to the other two complex-valued measurement matrices and outperformed the real-valued measurement matrices. Compared to the real-valued matrices, the average PSNR of the complex-valued Gaussian matrix improved by 2~3 dB. It even obtained a 4.35 dB boost on the “Woman” image. After Gram–Schmidt orthogonalization, the performance of the reconstructed image was greatly improved again.

**Figure 3:**
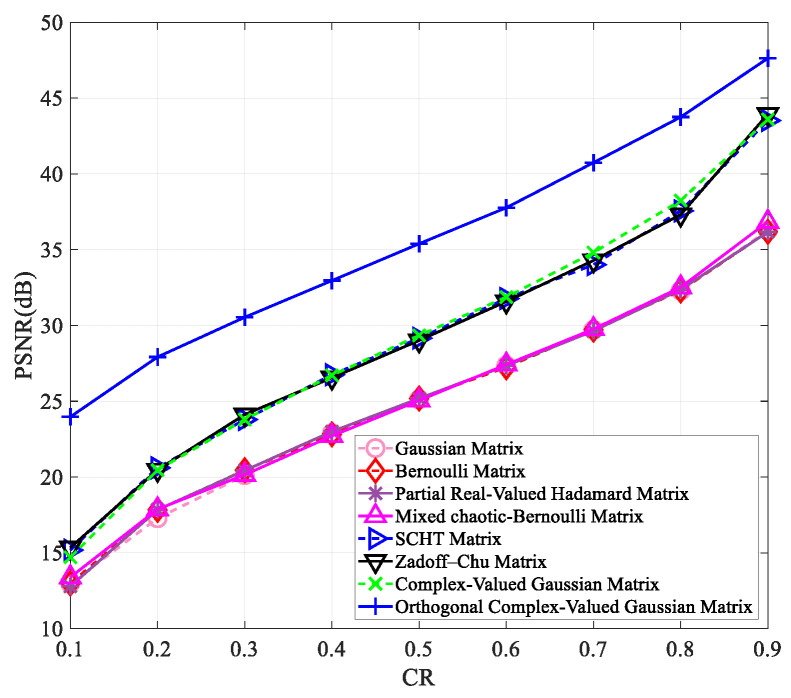
The PSNR values of the reconstructed “Peppers” image with the size of 256×256 using different measurement matrices. The first six measurement matrices in the figure are from references [9,10,11,17,19,27].

**Figure 4:**
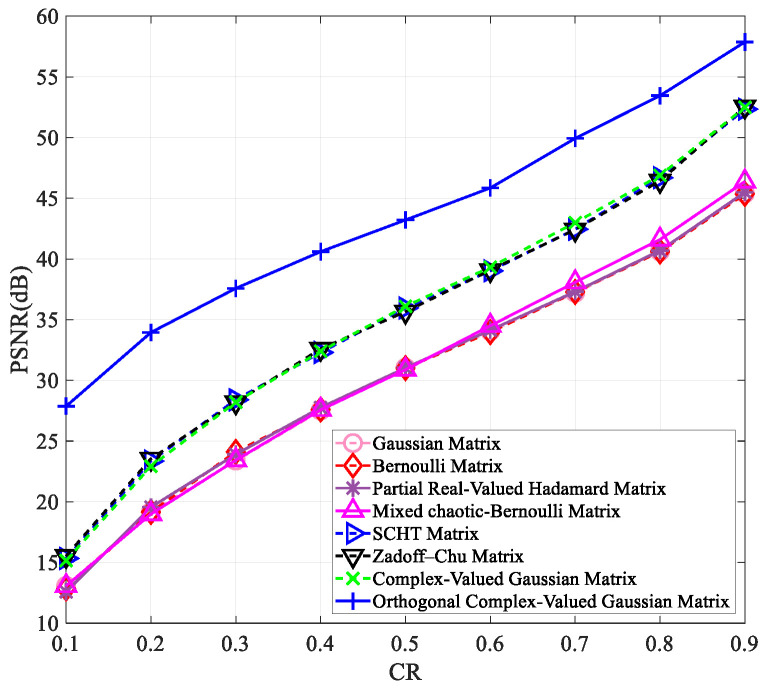
The PSNR values of the reconstructed “Woman” image with the size of 256×256 using different measurement matrices. The first six measurement matrices in the figure are from references [9,10,11,17,19,27].

**Figure 5:**
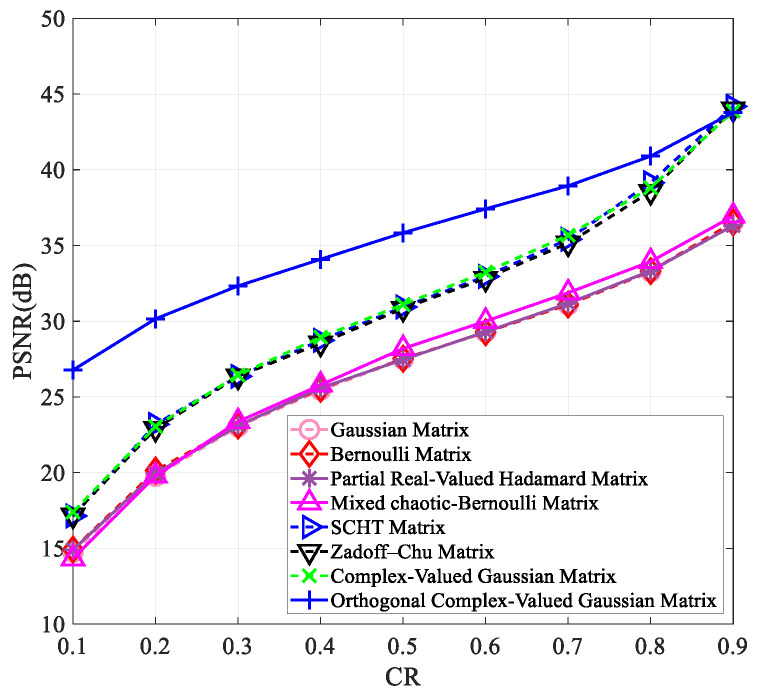
The PSNR values of the reconstructed “Peppers” image with the size of 512×512 using different measurement matrices. The first six measurement matrices in the figure are from references [9,10,11,17,19,27].

**Figure 6:**
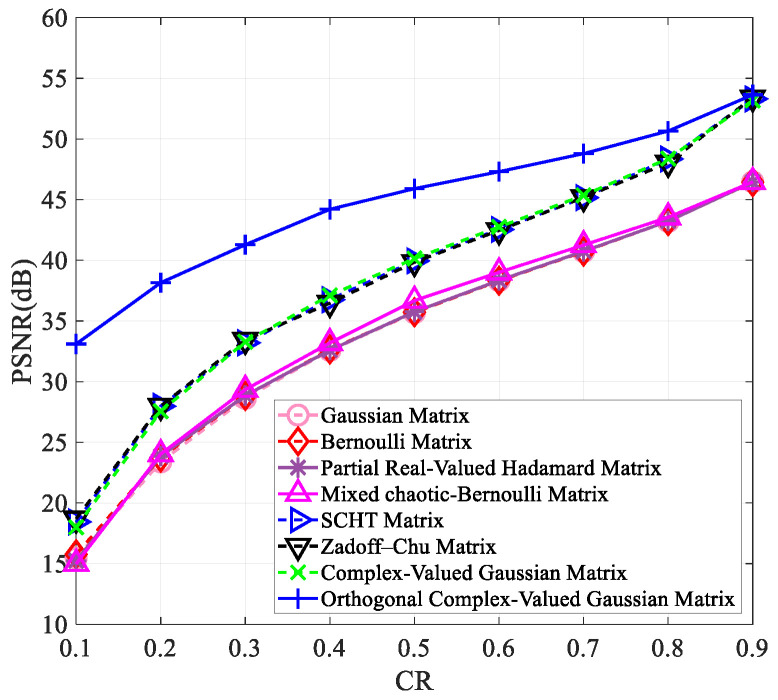
The PSNR values of the reconstructed “Woman” image with the size of 512×512 using different measurement matrices. The first six measurement matrices in the figure are from references [9,10,11,17,19,27].

To verify that our proposed measurement matrix has better reconstruction results than other matrices at varying compression ratios and image sizes, we tested it on images of “Peppers” and “Woman” with sizes of 256×256 and 512×512. The results are presented in Figure 3, Figure 4, Figure 5 and Figure 6.

Figure 3, Figure 4, Figure 5 and Figure 6 clearly show that all measurement matrices’ reconstruction quality steadily improves. For instance, Figure 6 illustrates that the reconstruction performance of various measurement matrices becomes better with an increase in compression ratio. The Gaussian matrix, Bernoulli matrix, and partial real-valued Hadamard matrices have very similar reconstruction performance, and the mixed chaotic–Bernoulli matrix has the best performance among these real-valued measurement matrices. The SCHT matrix and ZC matrix improve the reconstruction performance by approximately 3 dB compared to the previous four real-valued matrices. The complex-valued Gaussian matrix performs similarly to the SCHT matrix and ZC matrix. After Gram–Schmidt orthogonalization, the complex-valued Gaussian matrix has an improved reconstruction performance of about 5.7 dB on average compared to other complex-valued measurement matrices. Figure 3, Figure 4, Figure 5 and Figure 6 also show that the orthogonal Gaussian matrix has the best reconstruction performance, whether under the 256×256 size images or 512×512 size images.

### 4.2. Structural Similarity Analysis

In addition to PSNR, structural similarity [29] (SSIM) can also be used to evaluate the performance of a reconstructed image using the two measurement matrices. The value of SSIM ranges from 0 to 1, and the larger the value of SSIM, the better the quality of the reconstructed image. The calculation formula is as follows:

(13) $$\mathrm{SSIM}(a,b)=\frac{\left(2\mu_{a}\mu_{b}+c_{1})(2\sigma_{ab}+c_{2}\right)}{\left(\mu_{a}{}^{2}+\mu_{b}{}^{2}+c_{1})(\sigma_{a}{}^{2}+\sigma_{b}{}^{2}+c_{2}\right)}$$

Here, $\mu_{a}$ and $\mu_{b}$ represent the mean values of X and Y; $\sigma_{a}{}^{2}$ and $\sigma_{b}{}^{2}$ represent the variances of a and b; $\sigma_{ab}$ is the covariance of a and b; $c_{1}={\left(k_{1}\;L\right)}^{2}$ and $c_{2}={\left(k_{2}\;L\right)}^{2}$ are the constants used to maintain stability; L is the dynamic range of pixel values, and $k_{1}$ and $k_{2}$ are small constants.

**Figure 7:**
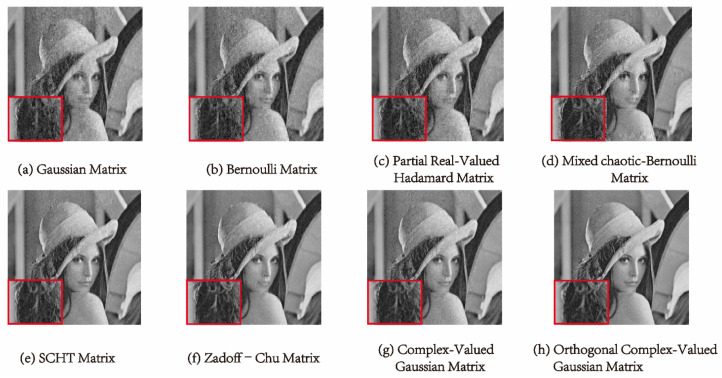
Comparison of reconstructed details of the “Lena” image with size 256×256 using different measurement matrices when the compression ratio is 0.5.

**Figure 8:**
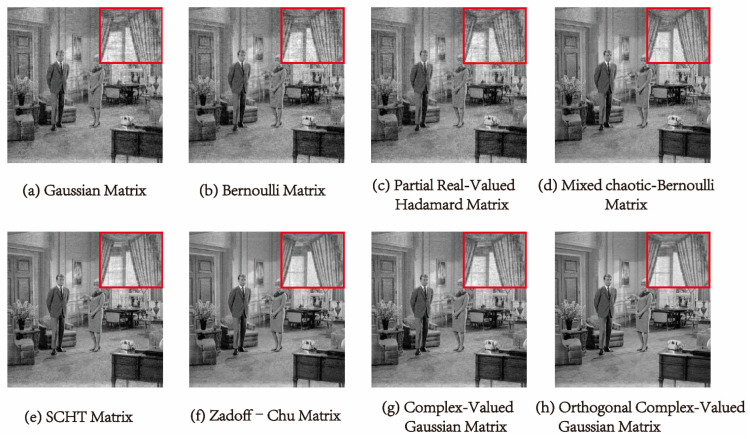
Comparison of reconstructed details of the “Livingroom” image with size 512×512 using different measurement matrices when the compression ratio is 0.5.

In Figure 7 and Figure 8, we can see the reconstructed images of “Lena” and “Livingroom” of 256×256 in size at a compression ratio of 0.5 using different measurement matrices. These measurement matrices can reconstruct the image well, but the reconstructed image using the measurement matrices shown in (a) to (d) appears fuzzy in the detailed part. In contrast, it is hard for the human eye to distinguish the details in the reconstructed image using the measurement matrices shown in (e) to (h). Therefore, we can assess the effectiveness of the measurement matrices by computing the SSIM value.

Table 3 displays the SSIM values calculated using Equation (13). For instance, when analyzing the “Lena” image, the first four measurement matrices yield SSIM values between 0.6 and 0.7. However, using the complex-valued Hadamard matrix, the SSIM value increases to 0.8378, and the ZC matrix further improves the value. Compared to the ZC matrix, the SSIM value of the orthogonal complex-valued Gaussian matrix proposed in this work is further improved, reaching 0.9731. The reconstruction performance of our measurement matrix is also optimal for several other images.

### 4.3. Comparison of Different Algorithms

**Figure 9:**
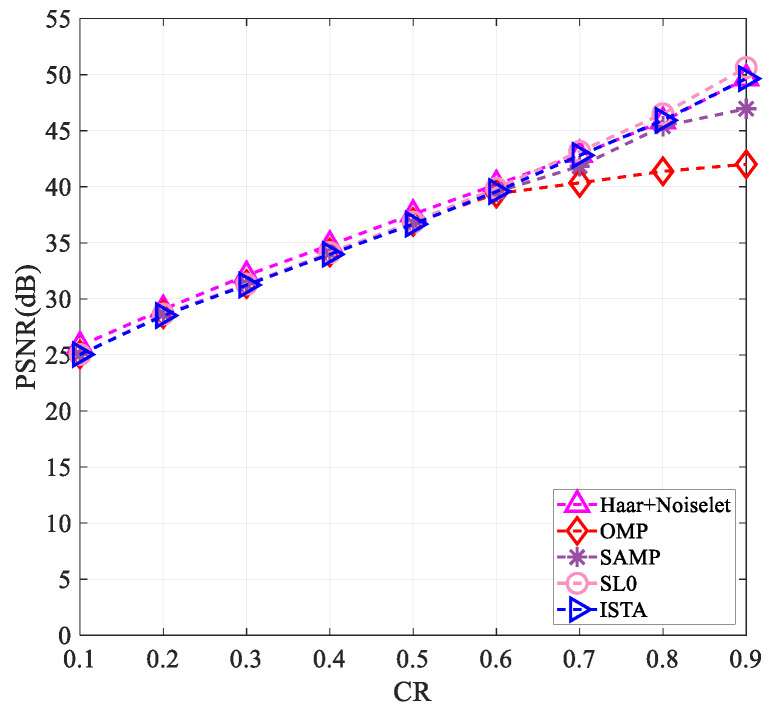
Performance comparison of reconstructed images using different algorithms. These five algorithms correspond to references [8], [20], [21], [22], and [23], respectively.

In this work, the Lena image with the size of 256×256 was used to verify the performance of the proposed orthogonal complex-valued Gaussian matrix under different reconstruction algorithms, such as SL0, OMP, SAMP, and ISTA. In addition, the recovery scheme based on the Haar wavelet transform with Noiselet transform, as in reference [8], was also considered, as shown in Figure 9. It can be seen that different reconstruction algorithms have almost identical performance when the compression ratio is lower than 0.6. For a higher compression ratio, the reconstruction performance of OMP becomes worse, while the other reconstruction algorithms still maintain good performance.

### 4.4. Sparsity Analysis of the Measurement Matrix

Since the use of a complex-valued measurement matrix would increase the computational complexity, for the sake of reducing the computational complexity in the measurement process and saving the storage space required for the measurement matrix storage, we performed a sparsification operation [30] on the measurement matrix. Firstly, we started by selecting a matrix of the same size as the measurement matrix and set all values to 1. For each column of the measurement matrix, d(d<<M) values were randomly reserved, and the values of the remaining positions were set to zero to obtain a sparse matrix. Then, the sparse measurement matrix was obtained by multiplying the measurement matrix with the corresponding position of the sparse matrix. This work explored the reconstruction performance of the “Lena” and “Cameraman” images of size 256×256 and 512×512 when the values of d are 3, 4, 8, 12, and 16, respectively.

**Figure 10:**
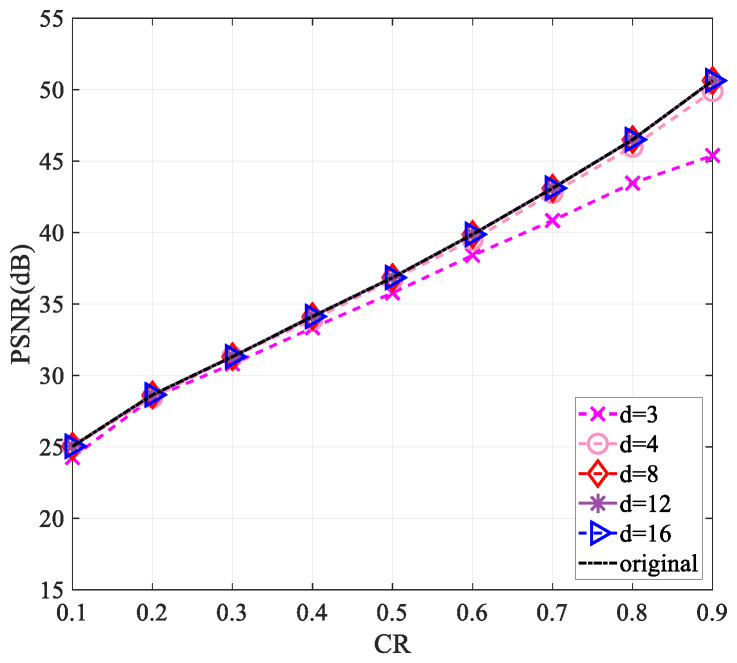
Impact of different sparsity on reconstruction performance using the “Lena” image of size 256×256.

**Figure 11:**
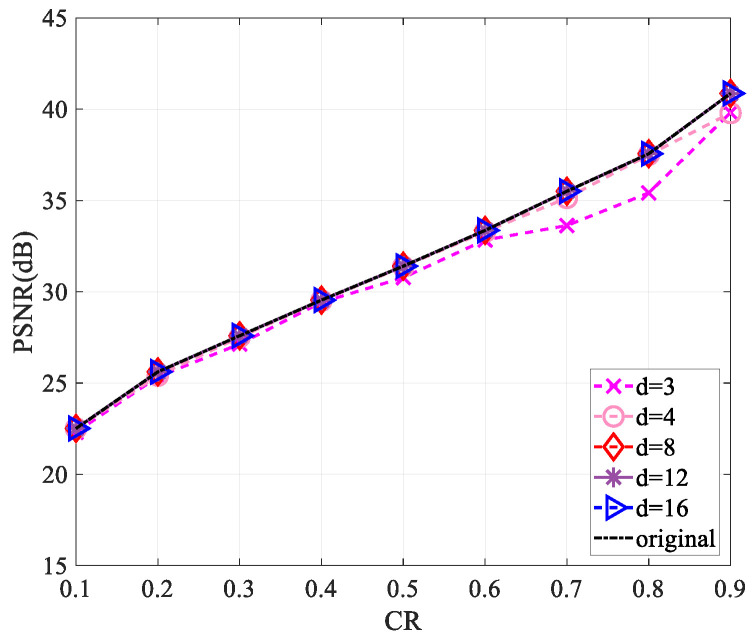
Impact of different sparsity on reconstruction performance using the “Cameraman” image of size 256×256.

**Figure 12:**
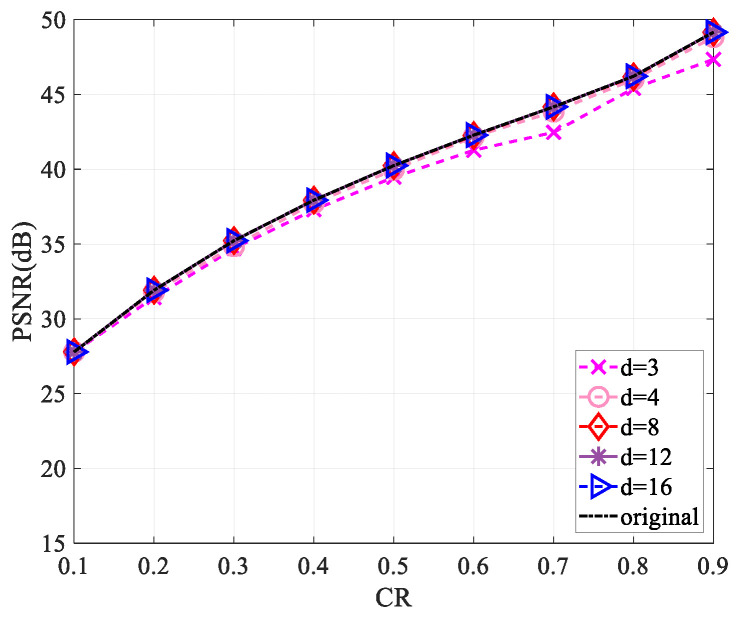
Impact of different sparsity on reconstruction performance using the “Lena” image of size 512×512.

**Figure 13:**
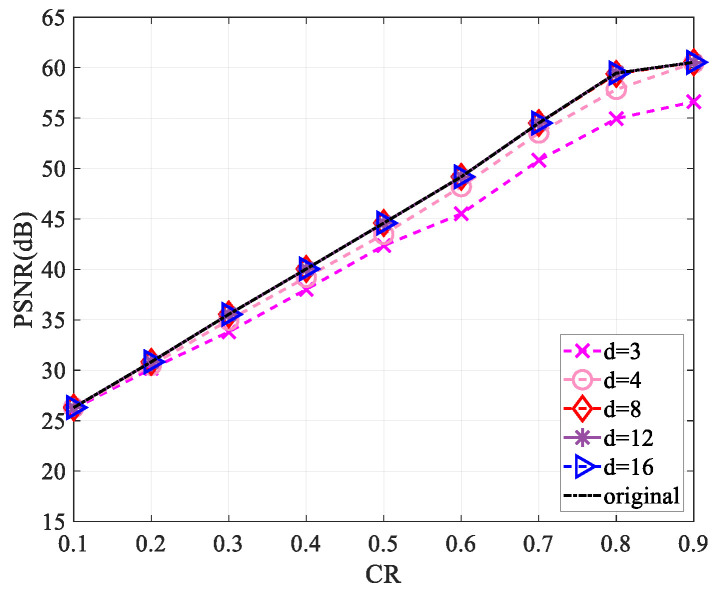
Impact of different sparsity on reconstruction performance using the “Cameraman” image of size 512×512.

Figure 10, Figure 11, Figure 12 and Figure 13 show that the largest performance loss occurs when d is set to 3. The reconstruction performance improves as the value of d increases to 4, 8, 12, and 16, with very minimal difference in the PSNR value. Compared to not using sparsification, the performance loss is less than 1 dB. Assuming the measurement matrix size is M×N(M=CR×M) and the image size is N×N, compressing the image requires 2×N×M×N multiplications and 2(N−1)×M×N+MN additions if the measurement matrix is without sparsification. Using the sparsified measurement matrix, when the sparsity is d(d<<M), only 2×N×d×N multiplications and 2×(N−1)×d×N+MN additions are needed when performing image compression, so a large part of the computational complexity is reduced. The loss of less than 1 dB in performance is acceptable relative to the reduced computational complexity. 

Table 4 shows the addition and multiplication complexity of several different matrices during the measurement process. In references [7,8], the Haar wavelet transform and the Noiselet transform were used to realize compression. For these two cases, the Haar wavelet transform only needs to multiply $\frac{1}{\sqrt{2}}$, which can be realized via a simple shift operation, and the Noiselet transform only needs addition operations. When using a partial Hadamard matrix for compression [11], no multiplication operation is needed because the partial Hadamard matrix is composed of 1 and −1, and the multiplication operation for −1 can be realized via a simple shift operation. For general real-valued measurement matrices, the matrix operations during compression require (N−1)MN addition operations and $MN^{2}$ multiplications, while the complex-valued measurement matrix requires twice the number of calculations. However, the computational complexity of the complex-valued measurement matrix after sparsification can be significantly reduced, and the performance loss after sparsification is entirely acceptable.

## 5. Conclusions

We proposed an orthogonal complex-valued Gaussian measurement matrix constructed by using two Gaussian matrices after Gram–Schmidt orthogonalization as the real and the imaginary parts, respectively. The simulation results demonstrate that using the complex-valued measurement matrix significantly enhances the reconstruction quality of images compared to using the real-valued measurement matrix. We then sparsified the measurement matrix and analyzed its calculation and reconstruction performance. The results show that the sparsified measurement matrix can effectively reduce the calculation amount with only a small loss in reconstruction performance. In addition, we compared the performance of our proposed orthogonal complex-valued Gaussian measurement matrix, real-valued measurement matrix, and other complex-valued measurement matrices on reconstructed images using different compression ratios and image sizes. The results show that the proposed complex-valued measurement matrix has better reconstruction effects. This work has implications for using complex-valued matrices as measurement matrices in compressed sensing.

## Figures and Tables

**Table 1:** When the compression ratio is 0.2, and under the image size of 256×256, the PSNR of the reconstructed image using different measurement matrices is compared. The value in bold is the best.

Measurement Matrix	Lena	Peppers	Woman	Boats
Gaussian Matrix [9]	19.15	17.28	19.16	17.78
Bernoulli Matrix [10]	19.32	17.83	19.16	17.88
Partial Real-Valued Hadamard Matrix [11]	19.33	17.84	19.56	17.85
Mixed Chaotic-Bernoulli Matrix [17]	20.23	17.86	18.99	18.51
SCHT Matrix [27]	22.03	20.61	23.35	20.90
Zadoff–Chu Matrix [19]	22.09	20.47	23.55	20.84
Complex-Valued Gaussian Matrix	22.01	20.46	22.93	20.59
Orthogonal Complex-Valued Gaussian Matrix	**28.64**	**27.92**	**33.95**	**29.12**

**Table 2:** When the compression ratio is 0.5, and under the image size of 256×256, the PSNR of the reconstructed image using different measurement matrices is compared. The value in bold is the best.

Measurement Matrix	Lena	Peppers	Woman	Boats
Gaussian Matrix [9]	26.30	25.12	31.04	26.42
Bernoulli Matrix [10]	26.25	25.18	31.01	26.56
Partial Real-Valued Hadamard Matrix [11]	26.34	25.18	31.03	26.56
Mixed Chaotic–Bernoulli Matrix [17]	26.52	25.05	30.88	26.94
SCHT Matrix [27]	30.40	29.16	35.96	32.09
Zadoff–Chu Matrix [19]	30.16	29.01	35.66	32.02
Complex-Valued Gaussian Matrix	30.49	29.34	36.10	32.33
Orthogonal Complex-Valued Gaussian Matrix	**36.85**	**35.38**	**43.21**	**42.23**

**Table 3:** Comparison of SSIM values of different reconstructed images when the original image size is 256×256, and the compression ratio is 0.5. The value in bold is the best.

Measurement Matrix	Lena	Peppers	Woman	Boats
Gaussian Matrix [9]	0.6628	0.6067	0.7943	0.6865
Bernoulli Matrix [10]	0.6667	0.6084	0.7965	0.6886
Partial Real-Valued Hadamard Matrix [11]	0.6674	0.6120	0.7987	0.6930
Mixed Chaotic–Bernoulli Matrix [17]	0.6817	0.6558	0.8335	0.7271
SCHT Matrix [27]	0.8378	0.7883	0.9232	0.8784
Zadoff–Chu Matrix [19]	0.8529	0.8296	0.9360	0.8936
Complex-Valued Gaussian Matrix	0.8235	0.7814	0.9191	0.8699
Orthogonal Complex-Valued Gaussian Matrix	**0.9731**	**0.9681**	**0.9879**	**0.9863**

**Table 4:** Comparison of process complexity of different measurement matrices.

Measurement Matrix	Addition Complexity	Multiplication Complexity
Haar + Noiselet [7]	$O(\frac{17}{2}MN+2MN\times \log_{2}(MN-1)-2\left(M+N\right))$	0
Haar + Noiselet [8]	$O(\frac{7}{2}MN+2MN\times \log_{2}(MN-1))$	0
Gaussian Matrix [9]	O((N−1)MN)	$O(MN^{2})$
Bernoulli Matrix [10]	O((N−1)MN)	$O(MN^{2})$
Partial Real-Valued Hadamard Matrix [11]	O((N−1)MN)	0
Mixed Chaotic–Bernoulli Matrix [17]	O((N−1)MN)	$O(MN^{2})$
SCHT Matrix [27]	O((2N−1)MN)	$O(2MN^{2})$
Zadoff–Chu Matrix [19]	O((2N−1)MN)	$O(2MN^{2})$
Complex-Valued Gaussian Matrix	O((2N−1)MN)	$O(2MN^{2})$
Orthogonal Complex-Valued Gaussian Matrix	O((2N−1)MN)	$O(2MN^{2})$
Sparse Orthogonal Complex-Valued Gaussian Matrix	O((2N−1)dN)	$O(2dN^{2})$

## Data Availability

The data presented in this study are available from the corresponding author upon request.
